# Morphological and Functional Alterations Induced by Two Ecologically Relevant Concentrations of Lead on *Danio rerio* Gills

**DOI:** 10.3390/ijms23169165

**Published:** 2022-08-15

**Authors:** Vittoria Curcio, Rachele Macirella, Settimio Sesti, Abdalmoiz I. M. Ahmed, Federica Talarico, Antonio Tagarelli, Marcello Mezzasalma, Elvira Brunelli

**Affiliations:** 1 Department of Biology, Ecology and Earth Science, University of Calabria, Via P. Bucci 4/B, 87036 Rende, Cosenza, Italy; 2 Natural History Museum and Botanical Garden, University of Calabria, Via P. Bucci 4/B, 87036 Rende, Cosenza, Italy; 3 Dipartimento di Chimica e Tecnologie Chimiche, University of Calabria, Via P. Bucci 12/C, 87036 Rende, Cosenza, Italy

**Keywords:** lead, zebrafish, gills, molecular biomarkers, morphological biomarkers, MTs, Na^+^/K^+^-ATPase, AQP3, SOD

## Abstract

Lead (Pb), due to its high toxicity and bioaccumulation tendency, is one of the top three pollutants of concern for both humans and wildlife and occupies second place in the Priority List of Hazardous Substances. In freshwater fish, Pb is mainly absorbed through the gills, where the greatest accumulation occurs. Despite the crucial role of gills in several physiological functions such as gas exchange, water balance, and osmoregulation, no studies evaluated the effects of environmentally relevant concentrations of Pb on this organ, and existing literature only refers to high levels of exposure. Herein we investigated for the first time the molecular and morphological effects induced by two low and environmentally relevant concentrations of Pb (2.5 and 5 μg/L) on the gills of *Danio rerio*, a model species with a high translational value for human toxicity. It was demonstrated that Pb administration at even low doses induces osmoregulatory dysfunctions by affecting Na^+^/K^+^-ATPase and AQP3 expression. It was also shown that Pb upregulates MTs as a protective response to prevent cell damage. Modulation of SOD confirms that the production of reactive oxygen species is an important toxicity mechanism of Pb. Histological and morphometric analysis revealed conspicuous pathological changes, both dose- and time-dependent.

## 1. Introduction

The intensive and continuous release into the environment of chemical pollutants is a direct consequence of economic development and human activities and has the potential to severely impact both humans and wildlife [1]. Aquatic habitats are especially affected due to water use in industrial processes, the deliberate or unintentional discharge of effluents from industry, agriculture, and domestic practices, and poor wastewater management [2]. In recent decades, environmental, economic, and social impacts of water pollution have grown steadily, making it clear that we are facing the most severe challenge globally [3]. Heavy metals have been classified among the most critical pollutants and the priority chemicals that will be monitored to preserve the quality of aquatic environments and human health [4,5,6,7].

Lead (Pb) represents 0.002% of the Earth’s crust and is ranked as the second most harmful environmental element [8,9]. Due to its abundance, rapid and high bioaccumulation, and long biological half-life, it gained more attention among other heavy metals (i.e., cadmium, chromium, and nickel) and is of great concern in food safety debates (FSS) worldwide [10]. Pb enters aquatic habitats through natural (i.e., erosion and atmospheric deposition) or anthropogenic inputs such as agricultural, urban, or industrial wastewater [9,10,11,12], resulting in widespread contamination of many water bodies, with an estimated concentration in surface water of 0.05 to 566.2 mg/L worldwide [13,14,15]. Additionally, Pb in drinking water is the primary pathway of accumulation in the human body with life-threatening health effects [16]. A systematic overview of the literature provides ample evidence of Pb toxicity and detrimental effects induced in fish have been recently reviewed by Lee and colleagues [17], which identified bioaccumulation, oxidative stress induction, neurotoxicity, and immune response alterations as the main toxicity mechanisms. In fish, lead enters the body from the water, sediments, and food [18], but in freshwater species, the uptake mainly occurs through the gills [17].

Whatever the absorption pathway, gills also represent the organs in which lead mainly accumulates [19,20]. The gill apparatus of fish is a complex organ that plays fundamental roles in many physiological functions, including gas exchange, osmoregulation, excretion, water balance, and acid–base regulation [21]. In direct and continuous contact with the external medium, the gills also offer a large surface to dissolved aquatic contaminants [22], thus representing the main target organs of various aquatic pollutants [23,24]. Indeed, morphological and functional modifications of the gill apparatus provide a powerful tool for assessing the health status of aquatic organisms [24,25,26]. Considering the physiological importance of gill tissue and its key role in Pb uptake and accumulation, it is surprising that a limited number of studies examined the effects of lead on this organ [27,28,29,30,31]. Most importantly, available data refer to high Pb levels of exposure, and there is a lack of experimental results on the effects of Pb environmentally relevant concentrations.

For decades, zebrafish has been employed as a non-mammalian model in revealing toxic mechanisms of chemicals and predicting environmental hazards [32,33]. Small body size, short reproductive cycle, high fecundity, and easy husbandry are among the reasons zebrafish became a popular laboratory animal for ecotoxicological studies [34,35,36]. Moreover, high physiological and genetic homology with humans provides the basis for the translational value of toxicity results from this model [34]. In adult zebrafish, Pb exposure triggers several toxic responses on various body tissues and organs, including (i) neurotoxic and behavioral alterations [15,17], (ii) oxidative stress induction [37], (iii) liver function impairment [37], (iv) endocrine disorders [38], and (v) liver and gut histological modifications [39]. However, only a few studies documented the response of the gills in the zebrafish model [20,37,40].

Hence, in order to have a more comprehensive overview of the Pb toxicity mechanisms in fish, in this study, the toxic effects induced by two sublethal and environmentally relevant concentrations of Pb (2.5 and 5 µg/L) were investigated for the first time in the gills of zebrafish (*Danio rerio*). The doses have been chosen based on the concentrations of Pb found in aquatic ecosystems worldwide and, in particular, are included in the range of concentrations of Pb in surface waters [13,14,15]. The pathological effects were first evaluated through an in-depth histological examination, followed by a morphometric analysis, which allowed the quantitative evaluation of the most relevant dimensions of gills affecting the diffusion distance and gas exchange in fish [41]. Several toxicity pathways have been suggested for lead in fish and other vertebrate models. Therefore, to evaluate the functional disorders under experimental conditions, we used a real-time PCR (RT-PCR) to detect and quantify key enzymes correlated to specific Pb toxicity pathways in zebrafish gills in a second step. Na^+^/K^+^-ATPase and aquaporin-3 (AQP3) are expressed within key osmoregulatory tissues where they mediate the active transmembrane movement of water and selected ions between intracellular and extracellular fluids [42,43] and are widely recognized as valuable biomarkers in xenobiotics-induced osmotic stress [24,26,29,44,45]. In fish, protective pathways are activated to regulate heavy metal ions and prevent tissue damage [46,47], including metallothioneins (MTs) induction. MTs, a group of low molecular weight proteins, are involved in the homeostasis and detoxification of essential and non-essential metals due to their ability to bind metals by their array of cysteine residues and are often used as biomarkers for monitoring heavy metals exposure in both laboratory and field conditions [25,46,48,49]. Both in vitro and in vivo studies demonstrated that in fish, Pb exposure stimulated reactive oxygen species (ROS) production resulting in oxidative damage and increasing antioxidant responses [17,50]. An important group of enzymes plays the role of scavenger compounds against free radicals, including superoxide dismutase (SOD), which is considered the primary intracellular antioxidant defense against free radicals [51,52]. To the best of our knowledge, the study presented here is the first report focusing on the morphological, morphometric, and functional alterations induced by non-lethal Pb doses in zebrafish gills; moreover, given the high translational value of the experimental model, the present results would also contribute to the discussion regarding Pb toxicity in humans.

## 2. Results

### 2.1. Histology

#### 2.1.1. Control Group

The gills of *Danio rerio* show the typical organization of other freshwater teleosts, and only a brief general description will be provided in this article.

**Figure 1:**
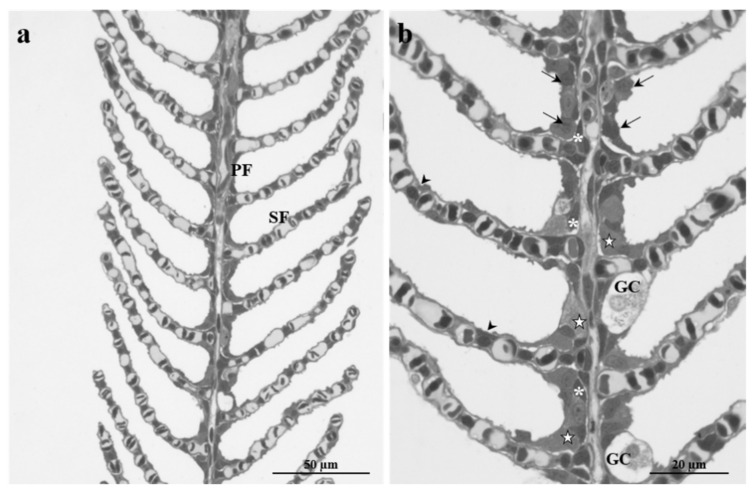
Light micrographs of *Danio rerio* gills in the control group. (**a**) Typical arrangement of the primary (PF) and secondary (SF) filament. (**b**) The primary epithelium is composed of pavement cells (arrows), goblet cells (GC), chloride cells (stars), and basal cells (asterisks). Note the pillar cells that control blood flow across secondary filaments (arrowheads).

Four gill arches support the gills giving insertion to a double series of filaments (primary filament), each of which gives rise to two series of lamellae (secondary filaments) (Figure 1a). The primary epithelium is composed of pavement cells (PVCs), which represent the most common cell type, and highly specialized cells: mucous cells or goblet cells (GC) and chloride cells (CC) (Figure 1b). The innermost epithelial layer is made by poorly differentiated basal cells (BC) in direct contact with the basal lamina (Figure 1b). CCs are characterized by a large number of mitochondria within their cytoplasm and are often distributed in clusters close to the onset of lamellae and in the interlamellar region of the filaments (Figure 1b). GCs, easily recognizable by their clear cytoplasm filled with secretory granules, are mainly distributed along the filament margin (Figure 1b). Respiratory lamellae are crossed by a network of capillaries delimited by pillar cells that regulate the blood flow (Figure 1b).

#### 2.1.2. Fish Exposed to 2.5 µg/L

**Figure 2:**
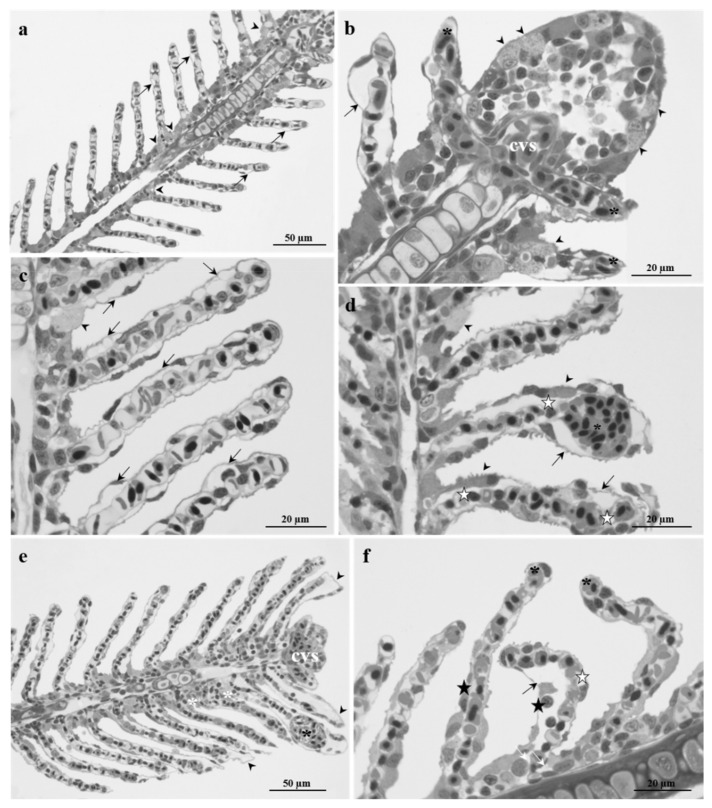
Light micrographs of *Danio rerio* gills after exposure to 2.5 µg/L of Pb. (**a**,**b**) After 48 h of exposure, hypertrophic chloride cells proliferate (arrowheads). Note the congestion of blood vessels (black asterisks) and central venous sinus (cvs). In the secondary lamellae, the lifting of epithelium is seen along with the dilation of the lamellar apical tips (arrows). (**c**,**d**) After 96 h, numerous chloride cells migrated into the secondary lamellae (arrowheads). The detachment of secondary epithelium is observed in almost all lamellae (arrows). Note the degeneration of pillar cells (white star) and aneurysms in the secondary filament’s apical portion (asterisks). (**e**,**f**) After 192 h, the main epithelium extended throughout the interlamellar area (white asterisks); wide intraepithelial lacunae are also detected (white arrows); note the congestion of the central venous sinus (cvs). Secondary lamellae are shortened and curved (white star), and the detachment of the secondary epithelium can also be reported (black arrows). Note degenerating cells (black stars), the enlargement of the lamellar apical tips (arrowheads), and aneurysms formation (black asterisks).

Gills exposed to the low Pb concentration showed many morphological alterations compared to control. After 48 h of exposure, the first modification observed in the primary filaments was the hypertrophy of CCs, which also proliferated, extending along the efferent borders of the primary filaments and the secondary lamellae (Figure 2a,b).

Blood vessels were congested, and this was particularly evident in the central venous sinus (Figure 2b). The most conspicuous alterations occurred in the secondary lamellae, where an extensive detachment of the epithelium from the underlying connective tissue was often observed with the appearance of intra-epithelial lacunae; a remarkable dilation of the lamellar apical tips was also evident (Figure 2a,b).

After 96 h of exposure, the lifting of lamellar epithelium becomes more severe and extensive, involving almost all secondary lamellae (Figure 2c,d). The phenomena of hypertrophy and proliferation of the CCs were pronounced, and it was possible to recognize numerous CCs scattered all along the secondary lamellae (Figure 2c,d). Moreover, the loss of organization of the vascular component was observed: the pillar cells were no longer distinguishable, and aneurysms in the apical portion of the lamellae were often seen along with diffuse vascular congestion (Figure 2c,d).

After 192 h of exposure to Pb, the morphological alterations became severe, involving the primary filament. A conspicuous proliferation of the primary epithelium was observed, which extends into the interlamellar space (Figure 2e). With further magnification, it was possible to note the presence of wide intercellular spaces, which at several points gave origin to intraepithelial lacunae (Figure 2f). It is also possible to observe the congestion of the central venous sinus of filaments (Figure 2e,f). The alteration of the secondary lamellae architecture was evident, and the filaments appeared shortened and folded (Figure 2f). Particularly severe were also the lifting of epithelium generating wide lacunae and the presence of degenerating cells (Figure 2e,f). The dilation of lamellar apical tips and the appearance of aneurysms were also noticeable (Figure 2e).

#### 2.1.3. Fish Exposed to 5 µg/L

**Figure 3:**
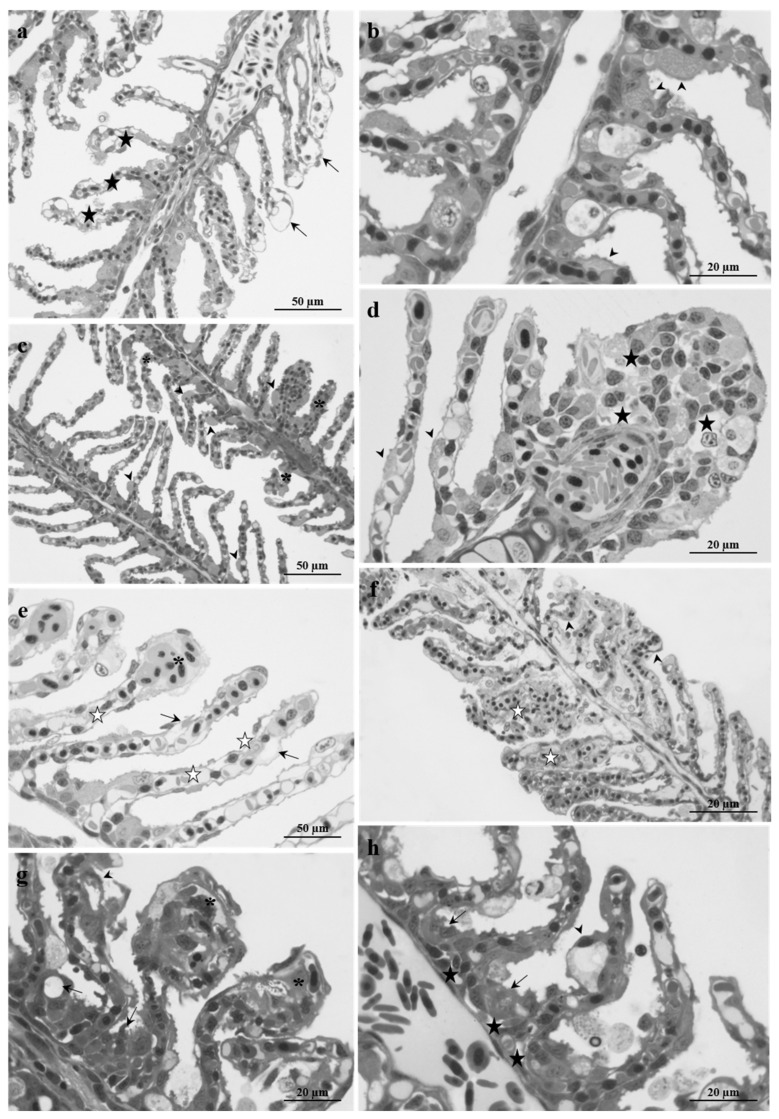
Light micrographs of *Danio rerio* gills after exposure to 5 µg/L of Pb. (**a**,**b**) After 48 h of exposure, the secondary lamellae are shortened and curved (black stars) and show dilated apical tips (arrows). Note hypertrophic CCs in the primary epithelium that also migrated to the proximal region of the secondary filament (arrowheads). (**c**–**e**) After 96 h in the primary epithelium, intercellular gaps and degenerating cells are observed (black stars). Note the shortened and folded lamellae and the presence of wide lacunae in the secondary filaments (arrows). White stars indicate vascular disorganization and pillar cell degeneration. Arrowheads indicated hypertrophic CCs that often migrated in the secondary lamellae. (**f**–**h**) After 192 h, secondary lamellae are curved (arrowheads), and the tissue proliferates, leading to lamellar fusion (white stars). Wide aneurysms are visible in the lamellar marginal tip (asterisks). Note intercellular gaps in the primary filament (black stars) and degenerating cells (arrows).

Histological analysis of the gills revealed an accentuated disruptive phenomenon starting from 48 h of exposure to the high lead concentration (Figure 3a,b). Secondary lamellae were especially affected, appearing shortened and folded with widely dilated apical tips (Figure 3a). The regular arrangement of blood capillaries was completely lost. Primary epithelium observations revealed proliferating and hypertrophic CCs that only cover the lamellae proximal region (Figure 3b).

The severity of these morphological changes increased after 96 h of exposure involving both the primary and the secondary filaments (Figure 3c–e). The hypertrophic CCs extended all along the secondary lamellae (Figure 3c,d), while intercellular gaps and degenerating cells became evident in the apical portion and the deep layer of primary epithelium (Figure 3d). The lamellae were further shortened and curved (Figure 3c). The secondary epithelium was always wrinkled and characterized by frequent lifting phenomena. The disorganization of the vascular component was accentuated, and the pillar cells were not distinguished (Figure 3e).

With prolonged exposure times, the histological alterations dramatically increased, and the typical arrangement of gills is compromised (Figure 3f–h). After 192 h of exposure, degenerative phenomena occurred in both primary and secondary epithelium. The secondary lamellae appeared strongly curved, and the tissue rose and proliferated, giving origin to large areas of lamellae that fuse together (Figure 3f).

Moreover, lamellae were affected by the appearance of large and frequent aneurysms, and the epithelial cells showed the pale cytoplasm of degenerating cell events (Figure 3g,h). An enlargement of intercellular spaces and cell proliferation were evident in the primary epithelium, along with the occurrence of degenerating cells (Figure 3h).

### 2.2. Morphometric Analysis

**Figure 4:**
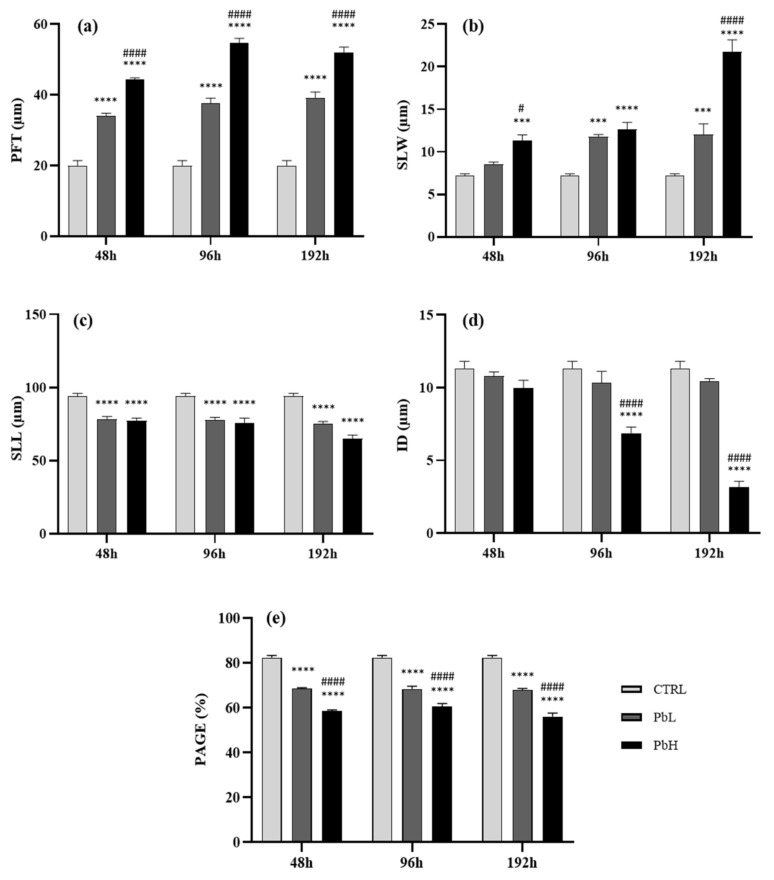
Morphometric parameters (mean ± SEM) in *Danio rerio* gills after exposure to 2.5 and 5 µg/L of Pb for 48, 96, and 192 h: (**a**) Primary filament thickness (PFT), (**b**) Secondary lamellar width (SLW), (**c**) Secondary lamellar length (SLL), (**d**) Interlamellar distance (ID), (**e**) Proportion of area of the secondary lamellae available for gas exchange (PAGE). Asterisks indicate significant differences between the treated and control groups. Hashtags indicate significant differences between the high Pb concentration group and the low Pb concentration group. *** *p* ≤ 0.001; **** *p* ≤ 0.0001; # *p* ≤ 0.05; #### *p* ≤ 0.0001.

Primary filament thickness (PFT)—Lead exposure induced an increase in the thickness of the primary epithelium. The increase in the thickness of the primary epithelium was observed at all time points starting from 48 h of exposure to the low Pb concentration compared to the control (Figure 4a). The same trend is observed in the specimens exposed to the high concentration, which showed a significantly increased epithelial thickness compared to the control and the low concentration groups at all time points (Figure 4a).

Secondary lamellar width (SLW)—Lead exposure induced an increase in the width of the secondary lamellae. The increase in the group exposed to low Pb concentration became significant compared to the control after 96, further increasing after 192 h (Figure 4b). In animals exposed to high concentration, the lamellar width was significantly greater than the control after 48 h and raised after 192 h reaching a maximum value (Figure 4b).

Secondary lamellar length (SLL)—Lead exposure induced a significant decrease in the length of the secondary lamellae in all exposed groups compared to the control. The minimum value was reached after 192 h of exposure to the high Pb concentration (Figure 4c).

Interlamellar distance (ID)—Exposure to the low Pb concentration induced a slight decrease in the interlamellar distance starting from 96 h of exposure which is not statistically significant compared to the control (Figure 4d). Instead, a significant reduction in the interlamellar distance was noticed in samples exposed to the highest concentration compared to both control and low concentration groups. The decrease was further accentuated after 192 h reaching the minimum value (Figure 4d).

Lamellar surface area for gas exchange (PAGE)—The lamellar tissue for gas exchange revealed a significant reduction in all groups exposed to lead at all time points compared to control. The decrease was more intense when a high dose was administered, and a significant difference was noticed between low and high Pb concentrations groups at all exposure times (Figure 4e).

### 2.3. Real-Time PCR

**Figure 5:**
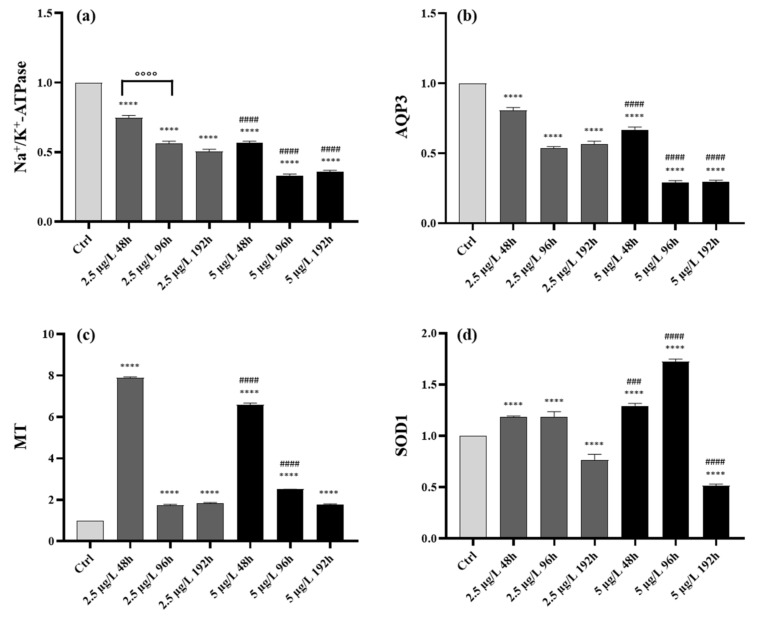
Gene expression (mean ± SD) in *Danio rerio* gills after exposure to 2.5 and 5 µg/L of Pb for 48, 96, and 192 h: relative mRNA expression of (**a**) Na^+^/K^+^-ATPase (*atp1a1a.1*), (**b**) AQP3 (*aqp3a*), (**c**) Metallothioneins (*mtf1*), (**d**) Superoxide dismutase (*sod1*). Asterisks indicate significant differences between treated and control groups. Hashtags indicate significant differences between high concentration and low concentration groups. Circles indicate significant differences between 96 h and 48 h treatment groups or 192 h and 96 h treatment groups. **** *p* ≤ 0.0001; ### *p* ≤ 0.001; #### *p* ≤ 0.0001; °°°° *p* ≤ 0.0001.

Na^+^/K^+^-ATPase (*atp1a1a.1*)—After 48 h of exposure to the low Pb concentration, the expression of Na^+^/K^+^-ATPase was significantly downregulated compared to the control group; after 96 h, a further decrease could be detected, and gene levels were significantly reduced compared to both control and 48 h exposed samples. The same trend was seen after 192 h when Na^+^/K^+^-ATPase reached the minimum expression level. The downregulation of Na^+^/K^+^-ATPase is more pronounced in the group exposed to the high concentration. The expression was significantly reduced at all exposure times compared to the control, and the minimum expression level was detected after 96 h of exposure. Additionally, a significant modulation was evident when comparing expression levels in animals exposed to low and high Pb concentrations at all time points (Figure 5a).

AQP3 (*aqp3a*)—In samples exposed to low Pb concentration, a significant decrease in aqp3a expression was detected at all time points compared to control, and the minimum level was reached after 96 h of exposure. A similar transcriptional response could be noticed when animals were exposed to the high Pb concentration, and also, in this case, the minimum value was found after 96 h. The modulation intensity was significantly higher than in control and low-Pb exposed groups (Figure 5b).

Metallothioneins (*mtf1*)—Exposure to the low concentration of Pb induced a significant upregulation of mtf1 compared to the control, peaking after 48 h of exposure. The expression level was drastically reduced after 96 and 192 h of exposure, still remaining significantly higher than the control. The expression pattern had the same trend in the samples exposed to the high concentration showing a maximum of expression after 48 h followed by a strong decrease with the continuation of exposure. The expression was significantly higher compared to control at all time points (Figure 5c).

Superoxide dismutase (*sod1*)—Exposure to the low concentration of Pb induces a significant upregulation of sod1 starting from 48 h of exposure compared to control; the transcription activity remained high after 96 h while it significantly decreased after 192 h compared to both control and earlier time points. A similar trend was detected in the group exposed to the high concentration, and the upregulation is followed by a significant reduction of expression level after 192 h. A statistically significant difference could be noticed compared to the control and the low concentration groups at all time points (Figure 5d).

## 3. Discussion

Due to their large surface, continuously in direct contact with the external medium, gills are recognized as a primary target organ of dissolved pollutants [22,23,24]. Moreover, since in freshwater fish, Pb is mainly absorbed through the gills, in which also the greatest accumulation occurs [19,20], it is not surprising that the first response to the presence of Pb would be an alteration of gill tissues. Despite this, to date, no studies evaluated the effects of environmentally relevant concentrations of Pb, and existing literature only refers to high levels of exposure. It must be emphasized that experiments examining concentrations well above tolerance limits do not represent real situations, and such biases may lead to failure in identifying risks posed under natural conditions.

In the present study, the responses of *Danio rerio* gills after exposure to two very low Pb concentrations at different time intervals were presented for the first time. The present findings, which document the high toxicity of this heavy metal even at very low doses, are of ecological interest since they fill a knowledge gap on Pb toxicity in fish, also contributing to fungicides risk assessment in the aquatic environment. Moreover, given the relevance of this model in translational toxicological research, the present data can be used to elucidate human-relevant toxicity mechanisms [53]. To accomplish their function as the main organ of gas exchange, the gills are highly perfused and provided with a large surface area and thin epithelia that facilitate the diffusion of gases [21]. However, such structural organization allows the passive exchange of water and ions, challenging osmotic and cell volume homeostasis (osmo-respiratory compromise) [54,55]. Considering the complexity of this multifunctional organ and its roles in many physiological functions, different and complementary tools must be employed to comprehensively assess Pb toxicity on gills. In this light, we evaluated histological and molecular targets providing evidence that Pb impairs *Danio rerio* gills and modulates the expression of genes involved in osmoregulation and the toxicological responses to heavy metals.

### 3.1. Morphological Modifications

Histological examination revealed conspicuous pathological changes whose severity increased with administered dose and exposure time resulting, at the end of the exposure period, in the high tested concentration in the complete loss of the gill arrangement. After exposure to both doses, the harmful effects first occurred in the respiratory lamellae, also involving the vascular component, and then spread to the primary filament. The lesions most frequently observed were: (i) hypertrophy and ectopia of CCs, (ii) detachment of the secondary epithelium, (iii) pillar cells degeneration, (iv) blood vessels congestion, and (v) shortening and curling of lamellae. Limited data exist for comparison with our results, as published papers only deal with high doses or long-term exposures. Still, observations on zebrafish gills are consistent with the few studies available reporting a similar pattern of alterations in other marine and freshwater species after exposure to Pb and other heavy metals [29,56,57].

In the present experiment, the prominent reaction of *Danio rerio* gills to the presence of Pb was the hypertrophy and proliferation of the primary epithelium and the pervasive inflammation with the consequent epithelial lifting of secondary lamellae. Both types of epithelial modification, while from different pathogenic origins, have the same purpose of preventing toxicant entry. These results agree with prevailing literature assumptions that the increasing diffusion barrier is an early defense mechanism against waterborne insult. There is clear evidence supporting this hypothesis, and epithelial lifting, hyperplasia, and hypertrophy have been reported in fish gills after exposure to aquatic pollutants both in the field and laboratory conditions ([29,58] and references therein).

The expansion of the primary epithelium observed here resulted in a significant increase in the thickness of the primary epithelium (PFT) and the width of the secondary lamellae (SLW), as clearly demonstrated by the morphometric analysis. Moreover, zebrafish exposed to Pb showed significantly shorter lamellae and a consequent reduction of surface available for gas exchange (PAGE). The requirement for increased blood flow to improve gas exchange, made difficult by epithelial modifications, induces secondary lamellae hyperemia and pillar cell alterations ([57] and references therein). Morphological alterations observed here, including primary epithelium proliferation and increased thickness aimed to counteract the pollutant intake, can function up to a certain threshold before the function of the gills is compromised as it hinders the gas exchange and osmoregulatory processes impairing the ability of the gills to mediate the active transmembrane movement of water and ions.

### 3.2. Gene-Expression

#### 3.2.1. Na^+^/K^+^-ATPase and AQP3

The gills of fish are essential to osmoregulation, a highly energetic process accounting for approximately 20–50% of total energetic expenditure [59]. Exposure to lead and other heavy metals induces osmoregulatory dysfunctions that are recognized as the main toxicity mechanisms of these pollutants in seawater and freshwater fish [20,29,60,61]. In basal conditions, Na^+^/K^+^-ATPase and aquaporin-3 (AQP3) are widely expressed within this key osmoregulatory organ and localize in the basolateral membrane of chloride cells (CCs), where they mediate the movements of water and selected ions between intracellular and extracellular fluids [42,43]. CCs are often a target of heavy metals’ toxicity [62], and in situations that prevent their normal function, the gills might promote their differentiation to increase ions uptake [62,63]. Our histological results confirm that exposure to Pb induces proliferation and ectopia of CCs, thus supporting the hypothesis that osmoregulatory disturbance stimulates a compensatory proliferative mechanism. However, the downregulation of Na^+^/K^+^-ATPase and AQP3 observed here more likely suggests a stereotypical mechanical defense function to reduce the intake of Pb than an attempt to improve osmotic exchanges. The responses of Na^+^/K^+^-ATPase were not consistent in different fish species when faced with Pb exposure, and in the gills, a decreased [29,61,64] or increased activity [65,66] have been documented. On the issue of AQP3, we are not aware of other reports concerning the modulation after exposure to Pb in freshwater species; still, the results presented here agree with our previous results on a seawater species [29].

#### 3.2.2. Metallothioneins (MTs)

In fish, the expression, transcription, and functioning of MTs are induced by a variety of essential and non-essential metals. Because of their ability to bind metals for detoxification, the increase in MTs levels is considered a protective response to prevent cell damage, and MTs are widely recognized as a reliable biomarker of heavy metal contamination under both laboratory and field conditions [25,46,47,48,49].

The molecular analysis clearly showed significant induction of MTs following Pb administration in *Danio rerio* gills in all experimental groups, according to previous reports on both freshwaters [67,68] and marine species [29]. Moreover, this result agrees with our previous study showing an MTs induction after lead exposure in embryo and larvae of the same species, thus demonstrating that zebrafish has a precocious ability to mount an adaptive response and maintain it into adulthood [8]. Interestingly, the upregulation observed here was more pronounced as early as 48 h of exposure in both low and high concentration groups and then decreased, still remaining higher than the control group. It seems that in gills, MTs act as an early defense mechanism since they minimize the bioavailability of Pb for other ligands, thus preventing heavy metals toxicity and increasing the resistance of tissues and cells. However, prolonged exposure to Pb excess reduces gills’ ability to synthesize MTs, as we demonstrated by histological observations, which show a strongly altered tissue after 96 and 192 h with obvious implications for protein biosynthesis. A similar pattern was observed in the gills of adult *Gobiocypris rarus* exposed to high Pb concentrations in which MTs expression significantly increased after 12 h of exposure and then gradually reduced [67].

#### 3.2.3. Superoxide Dismutase (SOD)

In fish, it has been widely established that exposure to heavy metals induces oxidative stress through the production of reactive oxygen species (ROS), widely recognized as a common toxicity mechanism driven by different environmental stressors. Excessive ROS production results in oxidative damage and increasing antioxidant responses to maintain the homeostasis of redox balance [17,50]. Teleosts utilize both enzymatic and non-enzymatic defense systems against free radicals, among which superoxide dismutase (SOD) plays the role of a first-line scavenger enzyme.

The Pb-induced ROS production and consequent upregulation of the antioxidant enzymes have been previously reported in several fish species, including zebrafish, at different developmental stages [40,47,69]. As shown by the experimental results, with increasing exposure time to Pb, the expression levels of SOD first increased and then decreased. The upregulation of SOD after 48 and 96 h can be explained by the need for gills to control the superoxide anion (O_2_^•−^) concentrations. However, the SOD-catalyzed dismutation reaction consumes a large amount of SOD, and with the prolonging of exposure to Pb, the activity of antioxidant enzymes is destroyed, thus inhibiting the expression of antioxidant genes [70,71,72,73]. In an experiment conducted on the same species, the level of sod1 in the gills exhibited a more delayed response, peaking after 72 and 96 h of exposure to Pb [40]. This discrepancy may be related to the different experimental conditions since the non-lethal concentration tested by Yin and colleagues [40] was much higher than those tested here (37.5- and 18-fold greater).

## 4. Materials and Methods

### 4.1. Fish Maintenance

For this study, 70 healthy wild-type zebrafish of both sexes aged 6–8 months were purchased from a local store. The animals were transported to the laboratory and maintained for two weeks in 100 L aquaria containing dechlorinated and aerated tap water at 26–28 °C and a light/dark cycle of 14 h/10 h (light on at 7.30 a.m.); water parameters were monitored daily and kept constant (pH 7.3, conductivity 300 Ls/cm, dissolved oxygen 8 ± 1 mg/L, hardness 180 mg CaCo_3_/L, absence of nitrate and nitrite). During the acclimatization period, the fish were fed twice a day with commercial food for tropical fish.

### 4.2. Test Substance and Selection Criteria for Pb-Exposure Concentrations

Two sublethal concentrations (2.5 and 5 μg/L) of lead acetate [Pb(CH_3_CO)_2_] (Sigma-Aldrich Chemical Co., Gillingham, UK) were tested. A stock solution of lead acetate (1000 µg/L) was prepared using distilled water; then, an appropriate amount of the stock solution was diluted in aged tap water to reach the selected concentrations. The determination of lead in water samples was carried out using an Elan DRC-e Inductively Coupled Plasma-Mass Spectrometry (ICP-MS) instrument (PerkinElmer SCIEX, Woodbridge, ON, Canada). Samples were added of 500 µL of ultrapure nitric acid and introduced into the instrumental system employing a PerkinElmer AS-93 plus autosampler and a cross-flow nebulizer with a Scott-type spray chamber. For the quantitative analysis, the calibration curve for the lead was built on five different Plasma–Mass in the calibration range of 0.1–50 μg/L. The analytical verifications of the actual concentrations were performed from time 0 every 24 h during the whole experiment (Table S1), and no noticeable variation was registered according to previous literature data [15,74,75,76]. The selection of doses is based on the Pb concentration range found in surface waters worldwide; therefore, the two concentrations are ecologically relevant [15]. Moreover, the two concentrations, 2.5 and 5 μg/L (low and high concentration, respectively), are in correspondence with 0.0015% and 0.0030% of the LC50–96 h value of adult zebrafish (171 mg/L) [77].

### 4.3. Experimental Design

Animals of comparable body dimensions were randomly assigned to the different exposure tanks equipped with aeration devices; each 30 L aquaria (40 × 32 × 20 cm) housed ten fish. The control group was kept in dechlorinated tap water. During the experiment, animals were maintained under the same conditions as before but fed every 48 h. A static exposure system was used following standard procedure guidelines with the renewal of test solutions after 24 h. The use of animals in this study was approved by the Institutional Animal Care and Use Committee at the National University of Entre Rios and the Italian University Institute of Rosario (Rosario, Argentina; protocol N°028/12). For each concentration, including the control, three replicates were performed. After 48, 96, and 192 h of Pb exposure, animals from both control and the exposure groups (*n* = 10) were deeply euthanized with tricaine methane sulphonate (20 mg/L MS 222 Sigma-Aldrich, St. Louis, MO, USA). Gill samples were excised and promptly cleaned of blood residues for subsequent molecular and morphological analyses. No mortality was recorded in both treated and untreated groups during the experimental period.

### 4.4. Light Microscopy

Samples were immediately fixed by direct immersion in 3% glutaraldehyde solution (Electron Microscopy Sciences, Hatfield, PA, USA) in phosphate buffer (0.1 M, pH 7.2) for 3 h at 4 °C and post-fixed for 2 h in 2% osmium tetroxide (ElectronMicroscopy Sciences, Hatfield, PA, USA) in the same buffer. The samples were then dehydrated in an increasing series of ethanol, soaked in propylene oxide, and embedded in Epon-Araldite (Araldite 502/Embed 812, Electron Microscopy Sciences, Hatfield, PA, USA).

Semi-thin sections (1–2 μm) were cut using a Leica UltraCut UCT (Leica Microsystems, Wetzlar, Germany), stained with toluidine blue (toluidine 1% in borate 2%), and examined under the light microscope (DM1000 LED; Leica Microsystems, Wetzlar, Germany) equipped with an Optika HDMI Digital Camera (Optika, Ponteranica, Italy).

### 4.5. Morphometric Analysis

Morphometric parameters were measured on semi-thin sections (toluidine blue-stained) using an image analysis program (NIH, Bethesda, MD, USA, developed at the National Institutes of Health, a part of the U.S. Department of Health and Human Services). For each animal of all treatment groups, including the control (*n* = 4), 5 gills photographs (40× magnification) were performed.

Several measurements were performed to evaluate the diffusion distance (gas exchange) in gills according to the method described by Nero and colleagues [78]. For each primary filament present in micrographs, primary filament thickness (PFT) was assessed; in detail, to obtain an average value for each filament, measures were taken in three different portions (two at the proximal, two at the medial, and two at distal portion). All secondary filaments were measured for length (SLL) and thickness (SLW), and interlamellar distance (ID); as we did for the primary filaments, thickness and interlamellar distance were measured at the proximal, medial, and distal portions of each secondary lamella to obtain an average value. Finally, the following formula was applied to calculate the area available for gas exchange: PAGE (%) = 100 × (mean SLL/(mean PFT + mean SLL).

### 4.6. RNA Isolation and Real-Time PCR

Total RNA (30 mg) was extracted from gills tissue of both treatment and control groups (*n* = 6) using the PureLink RNA Mini Kit (Thermo Fisher Scientific, Waltham, MA, USA) following the manufacturer’s instructions. The quality and quantity of RNA were checked spectrophotometrically using the NanoDrop One (Thermo Fisher Scientific, Waltham, MA, USA). Total RNA (2 µg) was used for first-strand cDNA synthesis employing the high-capacity RNA to cDNA kit (Applied Biosystems, Foster City, CA, USA); the obtained cDNA was stored at −20 °C.

The expression level of the following genes was evaluated: the ATPase Na^+^/K^+^ transporting subunit alpha 1a (*atp1a1a.1*, NCBI Reference Sequence NM_131686.1), the aquaporin 3a (*aqp3a*, NCBI Reference Sequence NM_213468.1), the metal-regulatory transcription factor 1 (*mtf1*, NCBI Reference Sequence NM_152981.1), and the superoxide dismutase 1 (*sod1*, NCBI Reference Sequence NM_131294.1). cDNA amplification was performed in a Light Cycler (Applied Biosystems StepOne, Real-Time PCR System, Foster City, CA, USA) using the TaqMan Gene Expression Assays (Thermo Fisher Scientific, Waltham, MA, USA) following the manufacturer’s instructions: one cycle at 50 °C for 2 min, 95 °C for 10 min, 40 cycles at 95 °C for 15 s, and 60 °C for 1 min. Each reaction contained 2 µL of cDNA, 10 µL of master mix (TaqMan Universal Master Mix II, Applied Biosystems), 1 µL of assay mix (TaqMan Gene Expression Assay), and 7 µL of RNase- and DNase-free water.

Each experimental unit, including the control, was replicated three times. The quantity of relative mRNA expression levels for all considered genes was normalized according to the average actin beta 1 expression level (*actb1*, NCBI Reference Sequence: NM_131031.2) following the 2^−ΔCt^ method [79].

### 4.7. Statistical Analyses

All statistical analyses were performed using Graph Pad Prism 8.00 (GraphPad Software Inc., San Diego, CA, USA) at a significance level of 0.05. Two-way ANOVA and Tukey’s multiple comparisons test were used to statistically compare significant differences in morphometric parameters and transcription levels between Pb-treated and control groups. The assumption of normality was tested using the Shapiro–Wilk test.

## 5. Conclusions

Overall, the present data demonstrate that short-term exposure to two very low and environmentally relevant Pb concentrations induces significant histological alterations in *Danio rerio* gills, whose severity increased with dose and exposure time. We showed that Pb exposure changes the most relevant dimensions of gills, thus affecting the diffusion distance and gas exchange. Pb administration at even low doses induces osmoregulatory dysfunctions by affecting Na^+^/K^+^-ATPase and AQP3 expression. Regulation of the osmotic permeability of the gills’ epithelium after exposure to lead and other heavy metals is an interesting topic that remains scarcely investigated; this study has partially increased our understanding, but more studies are needed to better clarify the pathway of toxicity of lead on the physiological function of gills. The metal ions that enter the gill cells immediately interact with the cytoplasmic components such as enzymes, compromising the main physiological functions. MTs upregulation shown here represents a first protective response to prevent cell damage confirming the role of MTs as a reliable biomarker of lead and other heavy metal contamination. Our results also confirm that the production of reactive oxygen species is an important toxicity mechanism of Pb.

## Data Availability

The data presented in this study are available on request from the corresponding author.

## Supplementary Materials

The following supporting information can be downloaded at: https://www.mdpi.com/article/10.3390/ijms23169165/s1.
