# Supplementary material for: Morphological and Functional Alterations Induced by Two Ecologically Relevant Concentrations of Lead on Danio rerio Gills
Source: Int J Mol Sci. 2022 Aug 15;23(16):9165. doi: 10.3390/ijms23169165 (PMC9409012; doi:10.3390/ijms23169165)
Supplement: Supplementary file 1 [file ijms-23-09165-s001.zip › ijms-1853815-supplementary.pdf]

Supplementary Files

Morphological and functional alterations induced by two eco-logically relevant concentrations of lead on Danio rerio gills

Vittoria Curcio [1]†, Rachele Macirella [1]†, Settimio Sesti [1], Abdalmoiz I. M. Ahmed [1], Federica Talarico [2], Antonio Tagarelli [3], Marcello Mezzasalma [1], and Elvira Brunelli [1*]

1 Department of Biology, Ecology and Earth Science, University of Calabria, Via P. Bucci 4/B, Rende (Cosenza) 87036, Italy

2 Natural History Museum and Botanical Garden, University of Calabria, Via P. Bucci 4/B, Rende (Cosenza) 87036, Italy

3 Dipartimento di Chimica e Tecnologie Chimiche, University of Calabria, Via P. Bucci 4/B, Rende (Cosenza) 87036, Italy

† Vittoria Curcio and Rachele Macirella contributed equally to this work and are the co-first authors.

* Correspondence: elvira.brunelli@unical.it; Tel.: +39-0984-492-996. m.mezzasalma@gmail.com; Tel.: +39-0984-492-976

Table S1. Detected Pb concentrations in the control group (Ctrl) and exposure solutions. The values are represented as mean ± SEM (n=9).

**Nominal concentration (μg/L)**	**Measured concentration (μg/L)**
Ctrl	0.03 ± 0.01
2.5	2.45 ± 0.06
5	4.93 ± 0.09
